# Online Machine Vision-Based Modeling during Cantaloupe Microwave Drying Utilizing Extreme Learning Machine and Artificial Neural Network

**DOI:** 10.3390/foods12071372

**Published:** 2023-03-23

**Authors:** Guanyu Zhu, G. S. V. Raghavan, Wanxiu Xu, Yongsheng Pei, Zhenfeng Li

**Affiliations:** 1 Jiangsu Key Laboratory of Advanced Food Manufacturing Equipment and Technology, School of Mechanical Engineering, Jiangnan University, Wuxi 214122, China; 2 Department of Bioresource Engineering, McGill University, 21111 Lakeshore Road, Sainte-Anne-de-Bellevue, QC H9X 3V9, Canada; 3 Key Laboratory of Urban Rail Transit Intelligent Operation and Maintenance Technology and Equipment of Zhejiang Provincial, Zhejiang Normal University, Jinhua 321004, China

**Keywords:** online machine vision, extreme learning machine, artificial neural network, moisture ratio modeling, adjustable power, cantaloupe microwave drying, image processing, computer vision, shrinkage

## Abstract

Online microwave drying process monitoring has been challenging due to the incompatibility of metal components with microwaves. This paper developed a microwave drying system based on online machine vision, which realized real-time extraction and measurement of images, weight, and temperature. An image-processing algorithm was developed to capture material shrinkage characteristics in real time. Constant-temperature microwave drying experiments were conducted, and the artificial neural network (ANN) and extreme learning machine (ELM) were utilized to model and predict the moisture content of materials during the drying process based on the degree of material shrinkage. The results demonstrated that the system and algorithm operated effectively, and ELM provided superior predictive performance and learning efficiency compared to ANN.

## 1. Introduction

Drying is an essential method in the global food-processing industry. By removing moisture from food materials, drying reduces water activity, decreases microbial growth, extends shelf life, reduces weight, and minimizes packaging costs [1,2]. Like many food-processing processes, the drying process is often conducted in a black box, where the dried materials cannot be observed or touched in real time [3,4,5]. Even in some drying vessels, such as hot air dryers or freeze dryers, where transparent windows are provided for observation, the precise properties of the samples, such as temperature, moisture content, and shrinkage, are not easily measured [6]. However, monitoring the properties of the materials during the drying process plays an important role in improving the quality of the final product [7,8]. For instance, it is crucial to adjust the drying rate based on the speed of shrinkage, reduce the drying power after the appearance of scorching, and determine the end point of drying [9,10]. For many years, scholars have been devoted to exploring methods for monitoring the quantity of the drying process [11,12,13,14].

The visual appearance of a material is often correlated with its internal physical properties. The application of machine vision makes it possible to capture the external characteristics of materials [15,16]. Machine vision refers to the technology that enables a computer system to acquire images or videos through a camera or other visual sensor and use computer algorithms for image processing and analysis [17,18]. In recent years, machine vision has been widely used in the food-processing industry and is gradually finding applications in the field of drying [19,20].

Initially, machine vision in food drying focused mainly on offline use, whereby an additional set of machine vision systems were set up outside the drying chamber. During the drying process, drying was interrupted at regular intervals to remove the materials from the chamber and place it in the vision system for information extraction before returning it to the drying chamber to continue the process. Onwude et al. monitored the color values of sweet potatoes during the drying process by using an offline vision system and found a good correlation between moisture content and color characteristics [21]. Lin et al. utilized hyperspectral imaging to detect the moisture distribution of four different vegetable samples during microwave vacuum drying, and established a feasible universal model [22].

The adoption of offline machine vision systems has provided us with a certain understanding of the physical appearance of materials during the drying process, which has positively contributed to the development of the drying discipline. However, the extraction–transfer–insertion operation is a slow process, and therefore the time interval between each operation cannot be too short; otherwise, the returned samples may not have sufficient time to heat up and complete the drying process. This means that the intermediate material states cannot be accurately understood during the experimental process; for example, in an experiment that collects data every 10 min, the material states at the second and eighth minute are unknown. Furthermore, the extraction operation breaks the continuity of the drying process, which implies that there may be differences in the drying state of the returned samples and the materials before extraction, as well as in the drying conditions inside the dryer, such as vacuum degree and hot air temperature. To tackle these issues, research on the online application of machine vision has emerged in some traditional drying methods, such as hot air drying [23,24].

Li et al. utilized online machine vision to measure the wrinkling and shrinkage of shiitake mushrooms during hot air drying and found that maintaining a higher relative humidity in the early stage of drying can inhibit the surface hardening of the materials, while rapid dehumidification in the later stage of drying can reduce shrinkage and wrinkling [25]. Raponi et al. employed a visual system embedded in a hot air dryer to capture organic apple sample images every 5 min throughout the drying process, enabling real-time monitoring of spatial and color changes [26]. Hosseinpour et al. performed online monitoring of color changes in shrimp during hot air drying and superheated steam drying, and investigated the effects of drying temperature and medium velocity on the kinetics of shrimp color change [27].

Most research on the application of machine vision for drying processes has been focused on nonelectromagnetic methods such as hot air drying, as the friendly environment of the drying chamber makes it easy to position machine vision components and obtain high-quality images. In these drying methods, cameras and light sources can be placed inside the drying chamber to achieve optimal imaging conditions. However, due to the characteristics of microwaves, all machine vision components must be placed outside the microwave drying chamber. The microwave cavity is encased in metal, and the window is covered with a dense metal mesh, making it challenging to obtain high-quality imaging that meets the required standards. As a result, there have been few studies on real-time machine vision in the context of microwave drying.

In addition, obtaining other physical properties of the materials being dried, such as temperature and moisture content, is also very difficult in microwave drying. The widely used thermocouple temperature measurement method in other drying processes is not applicable in microwave drying due to the presence of a metal probe. Electronic scales used to measure weight require certain weight conduction to perform measurements [28,29]. Moreover, in microwave drying the microwave oven that is widely used is equipped with functional components such as stirrers and magnetrons, which make it a huge challenge to arrange various measuring components in a limited space without interfering with each other, while ensuring adequate lighting and imaging conditions.

The artificial neural network (ANN) is a computer algorithm that simulates the workings of a biological neural network and is highly effective in describing nonlinear relationships between variables [30]. It has a wide range of applications in various fields, including the food-drying industry [31]. Liu et al. investigated the effects of drying conditions such as temperature on the color, vitamin C content, and rehydration ratio of dried kiwi slices. They utilized ANN modeling to evaluate the optimal drying conditions [32]. Fabani et al. applied ANN to model the drying curve of watermelon rind pomace under convective drying, and discovered that it provided better-fitting results than existing empirical models [33]. Liu et al. used ANN to successfully predict the energy and exergy parameters of mushroom slices during hot air impingement drying [34].

The extreme learning machine (ELM) is an improved neural network algorithm that has advantages over ANN. It is less prone to being trapped in local optima. In recent years, ELM has also been gradually applied in the food-processing field and has shown good performance in some studies [35,36]. However, there have been no studies found that utilize ELM with material shrinkage as input to model and predict water content during microwave drying processes.

The objectives of this article are as follows:

(1) to establish a microwave drying system based on online machine vision that includes a real-time machine vision unit, a real-time weight detection unit, and a microwave power automatic adjustment unit based on a real-time temperature monitoring unit;
(2) to develop an image-processing algorithm to extract material shrinkage features;
(3) to use ANN and ELM respectively, combined with online image-processing technology, to establish a model for the relationship between area shrinkage and moisture ratio of cantaloupes, and to verify the prediction effect of the model.

The novelty of this article was centered on the achievement of online machine vision monitoring during microwave drying, enabling real-time and uninterrupted monitoring of the materials’ appearance features. The moisture content was predicted by using ANN and ELM, meaning that the relationship between the appearance characteristics and internal physical quantities of the materials during the drying process was established.

## 2. Materials and Methods

### 2.1. Materials

The fresh cantaloupes (*Cucumis melo* var. *saccharinus*) were purchased at a local market. Only undamaged cantaloupes with clear skin lines were chosen for the experiments, which were stored at 5 °C and left at room temperature for 30 min prior to drying. The cantaloupes were peeled and sliced into 6 mm pieces by applying a mechanical cutter. These slices were further cut into cylindrical shapes with a diameter of 25 mm by using a cutting tool. To determine the initial moisture content, the cantaloupe samples were dried with hot air at 105 °C for 24 h, resulting in a moisture content of 9.87 g/g on a dry basis.

### 2.2. Experimental System

**Figure 1:**
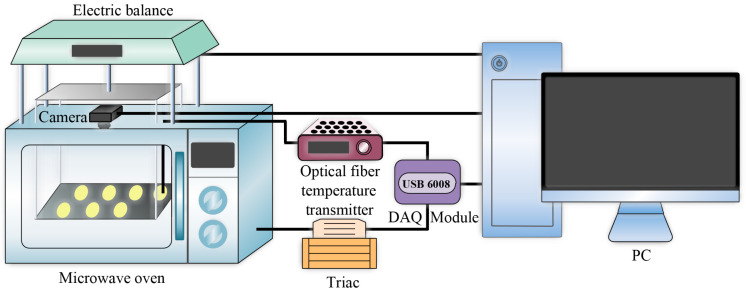
The schematic of the experimental system.

The developed experimental system consisted of three parts: online machine vision unit, variable-power microwave drying unit, and real-time weighing unit. The schematic of the system is illustrated in Figure 1.

#### 2.2.1. Online Machine Vision Unit

- Light Source

For machine vision systems, placing the light source inside the cavity can provide good lighting conditions. However, due to the special characteristic of microwaves, metal materials, such as wires, which are included in almost all kinds of light sources, cannot be put into the microwave cavity. Fortunately, the transparent window in the microwave door provides the possibility for light to enter the chamber without drilling more holes. Three LED light strips were therefore attached to the door of the microwave oven and proved to provide satisfactory light conditions for the experiments.

- Camera

Compared to the placement of the light source, choosing the location of the camera is more complicated, since it is impossible to find an acceptable nondestructive position on a microwave oven like the transparent window in the door. As a result, drilling is inevitable. There is a contradictory criterion for the size of the hole drilled to place the camera: the smaller hole contributes to lower microwave leakage. Nevertheless, the smaller the hole is, the less amount of light will enter the camera lens, resulting in worse image quality.

In order to determine the most appropriate hole size, a test experiment was conducted. Firstly, a 10 mm hole was drilled in the top of the inner cavity of the microwave oven. Secondly, a hollow round iron sheet was fixed above the hole. In each experiment, an iron sheet with different inner circle diameters of 4, 6, 8, and 10 mm was selected, respectively. Thereafter, drying experiments were conducted to test the image quality and the values of microwave leakage. It was found that the 6 mm hole can not only enable the camera to obtain the images that meet the requirements of subsequent processing, but also control the microwave leakage within the leakage standard. For stronger electromagnetic immunity and temperature resistance, an industrial camera was selected instead of an ordinary camera. The camera was positioned above the hole drilled in the top of the microwave oven and was able to produce clear images after being manually focused.

- Data Transmission

In terms of hardware, a USB cable was used to connect the industrial camera to the PC to transmit image data. In terms of software, two pieces of software—the Measurement and Automation Explorer (NI-MAX, Version 18.0; National Instruments Corporation, Austin, TX, USA) and LabVIEW (Version 16.0; National Instruments Corporation, Austin, TX, USA)—were utilized to configure the camera and capture images.

Setting appropriate values of camera attributes, such as exposure, white balance, etc., is crucial to obtain pictures with valid image information throughout the drying process, especially in confined spaces where adequate lighting is not available. NI-MAX provides a platform for adjusting camera parameters, in which images can be displayed instantly after adjustment, so that the optimal configuration can be acquired in a short time and saved for subsequent use.

The process of image acquisition and processing in this experiment had the characteristics of continuous acquisition, a large amount of data processing within a brief time, and a long duration. If the conventional image-processing programming scheme was followed, the memory would overflow after continuous collection, resulting in program termination. The Vision and Motion Module was used to allocate memory for the images before each acquisition, and release the memory after each processing session. In addition, the collected and processed data was written to the hard disk for permanent storage. Given that the computer was required to have sufficient processing time, the acquisition interval was set to 30 s.

#### 2.2.2. Variable-Power Microwave Drying Unit

Typically, microwave ovens are applied as pieces of laboratory microwave drying equipment. Nevertheless, original microwave ovens can merely provide fixed power, resulting in the inability to perform constant temperature drying. In this experimental system, a voltage regulation module was added, and the microwave oven circuit was modified, for the purpose of power control.

The core component for generating microwaves in a microwave oven is the magnetron. Within a certain range, the output power of the magnetron decreases with the reduction of the input voltage. According to the law, the microwave power can be adjusted by changing the input voltage of the microwave oven. Commercially available voltage regulators can adjust the main voltage, whereas the majority of them can just be regulated by a manual knob or input of voltage value, unable to realize continuous adjustment controlled by a PC. By employing a voltage-regulation module, whose functional part is a silicon-controlled rectifier unit, the above function can be achieved.

Provided that a microwave oven is plugged in at a voltage lower than the rated voltage, although the magnetron can work normally at low power, the internal fan and stirrer will cease to spin. Thus, the circuits of these two parts were separately connected to normal voltage to ensure their operation. Additionally, a reinforced fan was installed near the original exhaust holes on the left side of the microwave oven to remove moisture during the drying process. For the conversion of voltage signals and digital signals, a data acquisition (DAQ) module, NI USB-6008 (National Instruments Corporation, Austin, TX, USA), was utilized. With the DAQ module, DC voltage can be transformed into digital signals to be recognized by a PC, and 0–5 V voltage values calculated by a PC can be changed to real direct currents [37].

Traditional temperature-control systems, such as fixed-frequency air conditioners, utilize the two-position control law; that is, if the temperature is lower than the set temperature, the machine will be turned on, and on condition that it is higher than the temperature, it will be turned off. Although the design of this control strategy is simple, it has some disadvantages, such as low precision and large fluctuations. In this experiment, the PID control was adopted, which is based on feedback. With the PID strategy, the output power varied with the difference between the set value and the actual value as well as its differential and integral. Simply put, when the measured temperature was close to the set temperature, the output power would be reduced to a minor value to lessen temperature fluctuations. On the contrary, the output power would be increased to approach the set temperature faster, in the event that the temperature difference was large, especially in the initial stage of drying [29].

**Figure 2:**
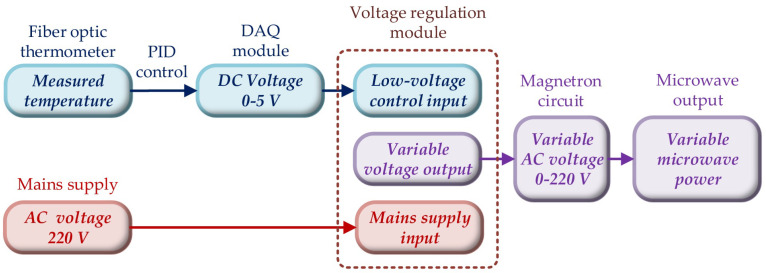
The implementation process of microwave power adjustment.

The implementation process of microwave power adjustment in terms of software and hardware is depicted in Figure 2. The real-time drying temperature of the materials was measured by the optical fiber inserted into a sample, and afterward converted into an electrical signal by the optical fiber thermometer and input into the PC. The corresponding 0–5 V DC voltage value was calculated by the PID control program in LabVIEW, and then input to the voltage regulation module through the DAQ module as the low-voltage control input. The other input terminal of the voltage-regulation module was connected to the city’s electrical grid, and the output end was capable of providing a variable AC power of 0–220 V. The main circuit of the microwave oven, namely the magnetron circuit, was linked to the output terminal to realize the adjustment of the microwave power.

#### 2.2.3. Real-Time Weighing Unit

Due to the presence of numerous metal components in the electronic scale, it could not be placed directly in the microwave oven. Therefore, nonmetal auxiliary components were required to facilitate weight transmission [38]. As shown in Figure 1, the electronic scale was placed above the microwave oven. The lower end of the electronic scale was connected to a metal rod, the other end of which was connected to a piece of Teflon board. Four Teflon rods were fixed at the four corners of the board. Four small holes were drilled in the top of the microwave oven to allow the four Teflon rods to pass through into the microwave oven. A material tray was connected below the rods to achieve the purpose of transmitting the weight of the samples to the electronic scale.

During the experiment, the electronic scale could measure the mass of the materials in real time and input the data to PC via an RS232-to-USB cable. After obtaining the mass $m_{t}$ (g) of the samples, the dry basis moisture content MC (g/g) during the drying process was calculated by Equation (1),

(1) $$MC=\frac{m_{t}-m_{d}}{m_{d}},$$

where $m_{d}$ refers to the dry mass of the materials.

In order to enhance the applicability of the model, normalization was conducted by utilizing the moisture ratio $MR_{t}$ to quantify the moisture content of the samples. The calculation equation is given below,

(2) $$MR_{t}=\frac{MC_{t}}{MC_{0}},$$

where $MC_{0}$ represents the initial moisture content and $MC_{t}$ denotes the moisture content at time *t* during the drying process.

### 2.3. Experimental Procedure

Before conducting the microwave drying experiment, several preliminary experiments were performed. Considering the drying time and quality of the dried product, the drying temperature of the materials was ultimately set to 60 °C [39]. The temperature was collected in real time and the microwave output power was continuously adjusted through the abovementioned PID strategy to achieve a constant drying temperature. The microwave drying process was stopped when the dry basis moisture content reached 0.176 (corresponding to a wet basis moisture content of 15%). Image processing and weighing operations were carried out every 0.5 min, and the processed data was recorded. The experiments were repeated three times, and the experimental data were averaged. After the experiment, a set of models were constructed by using ELM and ANN to predict moisture content based on shrinkage rate. Another drying experiment was conducted by using the obtained models for prediction, and the predicted values were compared and analyzed with the measured values to verify the effectiveness of the models.

### 2.4. Image-Analysis Algorithm

**Figure 3:**
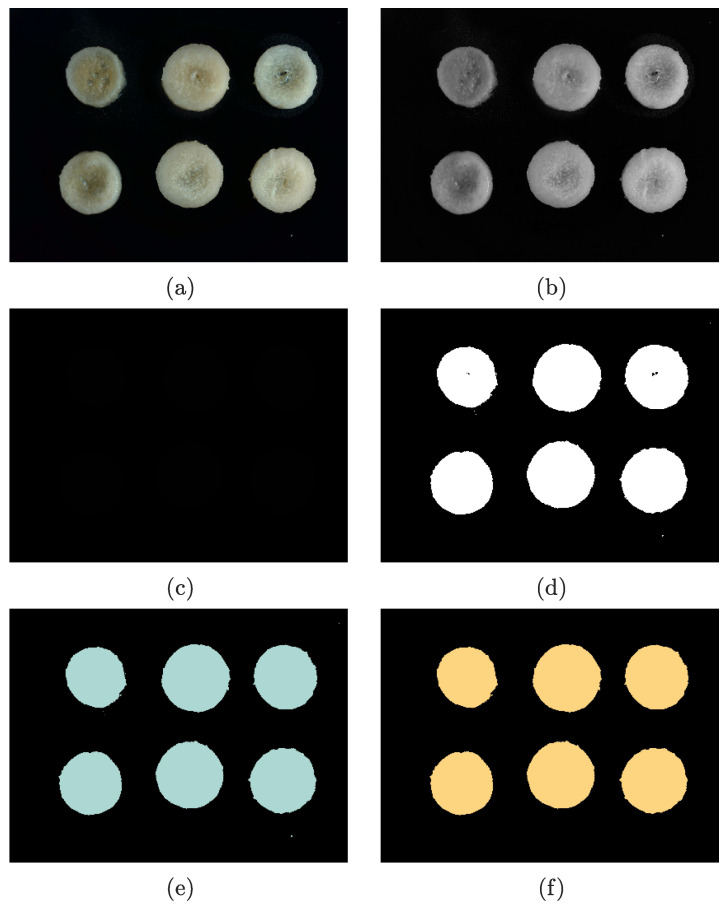
The steps of image processing: (**a**) original image; (**b**) grayscale image; (**c**) binary image; (**d**) converted black-and-white binary image; (**e**) image after filling holes; and (**f**) filtered image.

An image-analysis algorithm was developed to monitor the shrinkage of the materials. The algorithm included image acquisition, image processing, feature extraction, calculation, and recording. A typical example of image processing steps is illustrated in Figure 3.

#### 2.4.1. Image Acquisition and Grayscale Processing

The image captured by the camera is an RGB image (Figure 3a), in which each pixel contains 24-bit data, that is, 8-bit data for each of the red, green, and blue channels. Since each picture contains too much data, on condition that the image-processing program is carried out directly, the computer CPU will be overloaded, and the process will be time-consuming. As a consequence, it is necessary to reduce the data dimension of the collected pictures.

Considering that the area of the sample has no correlation with their color, the color information can be removed to realize the first step of data dimensionality reduction. The background in this machine vision system is black, meaning that the color values of the pixels in the background part are 0 or close to 0. On the contrary, the high contrast between the sample and the black background brings the color values of the materials well above 0, allowing the color image to be grayed out with the mean method. The R, G, and B components of each pixel extracted from the original image were averaged and subsequently synthesized into a pixel matrix, which was further converted into a displayed grayscale picture (Figure 3b).

#### 2.4.2. Image Binarization

The color information of each pixel was reduced from 24 bits to 8 bits after applying the grayscale algorithm. If 1-bit data could be employed, that is, 0 (black) represents the background, and 1 (white) stands for the samples, the data volume of the pictures would be greatly compressed. Since the target and the background occupy different grayscale ranges in the collected pictures, the means of threshold segmentation is suitable for outputting 1-bit images (binary images), so as to achieve the second step of data dimension reduction. Throughout this experiment, the metric approach was used to calculate the optimal threshold of each image.

The metric thresholding method computes a value for the respective threshold that is determined via the surfaces representing the initial grayscale. The ideal threshold is the value *k* at which the expression below takes the minimum value,

$$\sum_{i=0}^{k}h\left(i\right)|\left(i-\mu_{1}\right)|+\sum_{i=k+1}^{N-1}h\left(i\right)\left|\left(i-\mu_{2}\right)\right|,$$

where *k* is the gray-level value chosen as the threshold such that all gray-level values less than or equal to *k* belong to one class 0 and the other gray-level values belong to another class 1, *i* represents the grayscale value, and h(i) stands for the number of the pixels at every grayscale, *N* means the total figure of gray levels, which is 256 for an 8-bit picture, *n* indicates the sum of the pixels, and $\mu_{1}$ is the mean of all the pixel values that lie between 0 and *k*, and denotes the average number of the total pixel values in the range of k+1 to 255.

The binary image is shown in Figure 3c. For the image display system of LabVIEW, binary images can only be displayed and saved as 8-bit grayscale pictures. When the values 0 and 1 in binary pictures are taken as gray values, both of them are close to black. For the purpose of black-and-white image output, the value of each pixel was multiplied by 255. The converted binary image is displayed in Figure 3d.

#### 2.4.3. Particle Recognition and Hole Filling

As can be seen from the binary picture (Figure 3d), the region of interest (samples) has been distinguished from the background. Nevertheless, due to the rapid rate of microwave drying, the moisture moves and evaporates quickly, resulting in the formation of air bubbles. The bubbles may produce specular reflection, making the corresponding part of the image relatively dark and potentially identifiable as the background color after binarization. The preliminary experiment showed that with the generation and disappearance of the bubbles in different positions of the materials, the processed data demonstrated a fluctuation, leading to a decrease in data reliability. Hence, measures must be taken to eliminate the part of the bubbles in the image. The algorithm of particle-filling holes was adopted in this experiment.

A particle is a concept in machine vision referring to the area composed of interconnected nonzero pixels in an image. Particles are usually target areas, which are identified as foreground states through previous processing methods such as threshold segmentation. The samples can then be analyzed as particles. A particle hole is another concept, meaning a continuous area of zero-valued pixels completely encircled by nonzero pixels. The air bubbles that appear during the drying process can exactly be regarded as particle holes.

The algorithm of particle-filling holes was utilized to stuff the holes detected in a particle. The holes were filled with the pixel value of 1. After being processed by the algorithm, the internal pattern noise of the materials was removed. In order for the new picture to be differentiated from the previous ones, the foreground color was switched to light blue. The completed filled-particle image is shown in Figure 3e.

#### 2.4.4. Particle Filtering

After extracting the particles, it was found that the total number of the particles exceeded 1000. The trigger is that impurities, such as dust on the background cloth, caused numerous tiny particles outside the target particles to be captured, increasing the effective particle pixels. Since the pixel numbers of tiny particles are far fewer than those of large particles that represent the materials, effective particles can be filtered if a pixel threshold is selected, above which the particles are retained. The filtered image is displayed in Figure 3f with the particle color converted to yellow.

#### 2.4.5. Image Feature Extraction and Shrinkage Quantification

After the conversion, enhancement, and processing of the picture, the specific image feature needs to be extracted for the data of interest. Given that the research object of this experiment is the shrinkage ratio of the materials, it is dispensable to measure the actual area of each sample. As a method, the area of the materials can be represented by the number of pixels. Thus, the total area of the samples *A*, namely the sum of the pixels, can be calculated by Equation (3),

(3) $$A=\sum_{j=1}^{u}P_{j},$$

where $P_{j}$ is the number of pixels of respective particles left after being filtered and *u* is the number of the materials.

As microwave drying proceeds, the samples undergo area shrinkage, which can be expressed by using the area ratio AR. AR at time *t* after the start of drying $AR_{t}$ was determined by Equation (4),

(4) $$AR_{t}=\frac{A_{t}}{A_{0}},$$

where $A_{t}$ is the total area of the materials at time *t*, and $A_{0}$ stands for the area at the initial time.

#### 2.4.6. Consecutive Collection, Processing, and Recording

**Figure 4:**
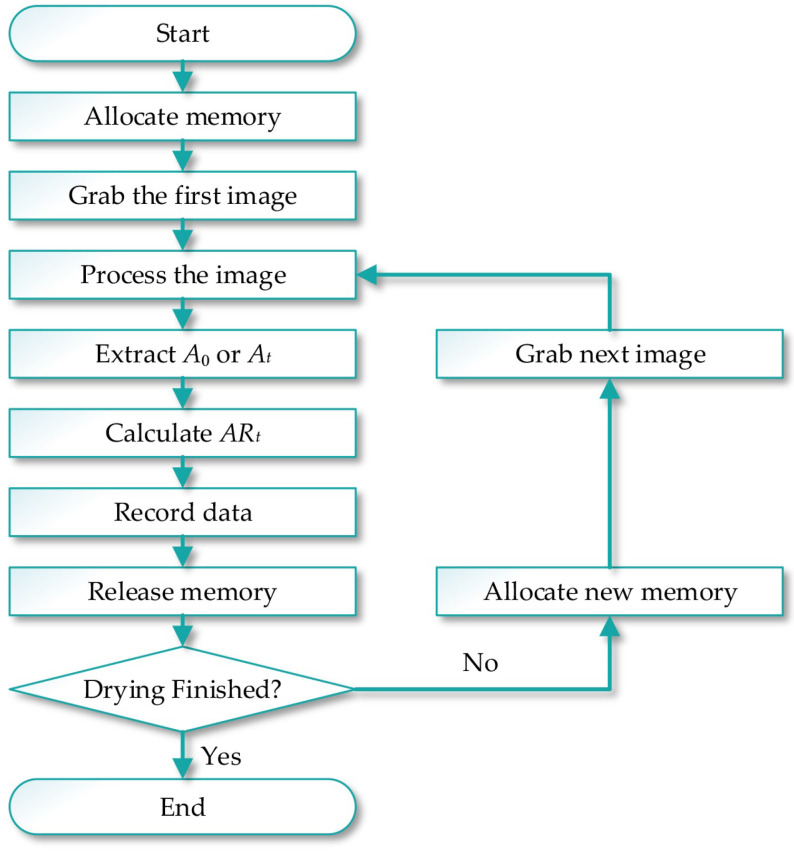
The flowchart of the machine vision part of the experiment.

The flowchart of the machine vision part of the experiment is shown in Figure 4. After the microwave drying started, the PC allocated memory for image acquisition and processing, and the camera captured the first image. After the collected picture was processed by the image-processing algorithm described above, $A_{0}$ was extracted and stored. Then, the memory was freed before new memory was allocated for the next image to avoid memory overflow. In the next step, the second picture was collected and $A_{t}$ was extracted. $AR_{t}$ was subsequently calculated and recorded. The cycle was repeated until the drying process ended.

### 2.5. Modeling and Prediction

#### 2.5.1. Artificial Neural Network

ANN is a nonlinear structure that can map any complex and dynamic phenomena by loosely imitating the actions of neurons in the human brain. With its high robustness, it can better and more practically reveal hidden relationships by applying pattern-recognition theory compared to traditional mathematical and statistical methods.

In this article, ANN was employed as one of the methods to establish a model between the shrinkage and moisture ratio of the samples. The input variable was the materials’ area ratio obtained through image-processing algorithms, while the output variable was the moisture ratio during the drying process. A feedforward neural network with one hidden layer was selected, and the Levenberg–Marquardt algorithm was utilized for training. This algorithm introduced a regularization term into the objective function, which enhanced the convergence and stability of the training process. The trained model was then employed to predict the outcomes of a new set of dry experiments, and the predictive performance of the model was evaluated.

#### 2.5.2. Extreme Learning Machine

ELM is a new learning algorithm used for training the single-layer feedforward network (SLFN). The hidden nodes in ELM are theoretically randomly initialized and fixed without requiring iterative updates [40,41]. As an optimization-free algorithm, it can train faster than traditional learning methods such as ANN or the backpropagation (BP) algorithm. Moreover, the ELM model can achieve similar generalization bounds as the conventional feedforward neural network (FNN) with commonly used activation functions. Its main feature is the random initialization of the weights and biases between the input and hidden layer, the calculation of the hidden layer output by using an activation function, and obtaining the optimal output weights through the analysis of the weight matrix and the calculation of the pseudoinverse matrix.

The training process of ELM typically includes the following steps.

(1) Input data and labels. ELM accepts input data and corresponding labels, where the input data can be vectors of any dimensionality and the labels can be discrete or continuous output values.
(2) Random initialization of weights and biases. ELM uses random initialization to set the weights and biases between the input and hidden layer, which are randomly generated and can be regularized to avoid overfitting.
(3) Calculation of the hidden layer output. ELM uses an activation function (such as sigmoid function, ReLU function, etc.) to calculate the output between the input and hidden layer. The number of nodes in the hidden layer is usually set by oneself, but it is necessary to ensure enough nodes to ensure the complexity and expressive power of the model.
(4) Analysis of the weight matrix. In ELM, the weight matrix is usually a randomly generated matrix, and its analysis method can be obtained by calculating the generalized inverse or pseudoinverse of the matrix. Through the analysis of the weight matrix, ELM can obtain the optimal output weight.
(5) Calculation of the output layer weight. By calculating the pseudoinverse matrix, the optimal weight of the output layer can be obtained, which is calculated by linear regression.
(6) Calculation of the final output. By using the optimal output weight and activation function of the output layer, the final output result can be calculated.

In this research, ELM was also utilized to model the relationship between the area ratio and moisture ratio of the materials during microwave drying. The input and output variables were set the same as ANN. The ELM algorithm was trained by using a training dataset, with sigmoid employed as the activation function. After manual tuning, the optimal number of hidden layers was determined to be 12. The same prediction dataset was used to compare the prediction results with ANN.

#### 2.5.3. Performance Indicators

To evaluate the performance of the ANN and ELM models, three metrics were used: root mean squared error (RMSE), mean absolute error (MAE), and coefficient of determination ($R^{2}$). The calculation methods for these metrics are presented below [42],

(5) $$RMSE=\sqrt{\frac{1}{n}\sum_{i=1}^{n}{\left({\hat{Y}}_{i}-Y_{i}\right)}^{2}},$$

(6) $$MAE=\frac{1}{n}\sum_{i=1}^{n}\left|{\hat{Y}}_{i}-Y_{i}\right|,$$

(7) $$R^{2}=1-\frac{\sum_{i=1}^{n}{\left(Y_{i}-{\hat{Y}}_{i}\right)}^{2}}{\sum_{i=1}^{n}{\left(Y_{i}-\bar{Y}\right)}^{2}},$$

where $Y_{i}$ represents the obtained moisture ratio for the *i*th measurement, ${\hat{Y}}_{i}$ denotes the predicted moisture ratio, $\bar{Y}$ is the mean value of moisture ratio for all measurements, and *n* is the number of measurements.

#### 2.5.4. Software

MATLAB (version 2019a, MathWorks, Natick, MA, USA) was utilized for programming, training, and predicting ANN and ELM models, as well as for computing performance indicators and generating statistical plots.

## 3. Results and Discussion

### 3.1. Modeling

**Figure 5:**
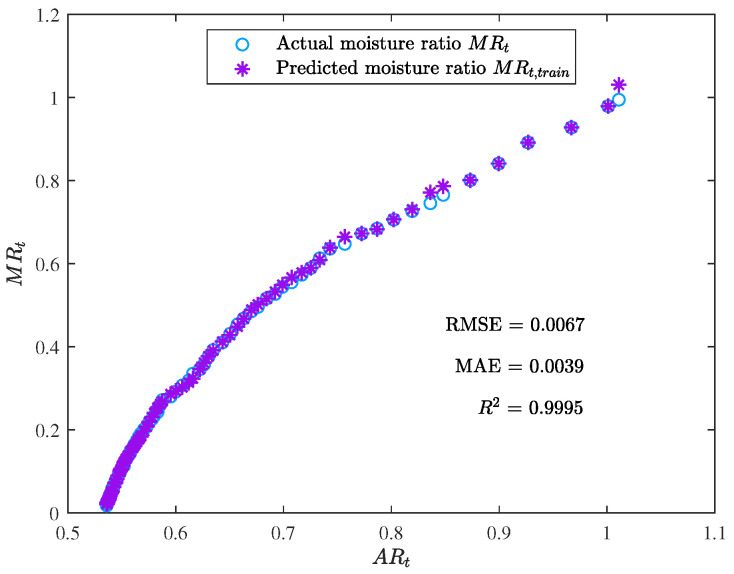
The training result of the ANN model (the moisture ratio given by the trained model $MR_{t,train}$ vs. the area ratio $AR_{t}$).

**Figure 6:**
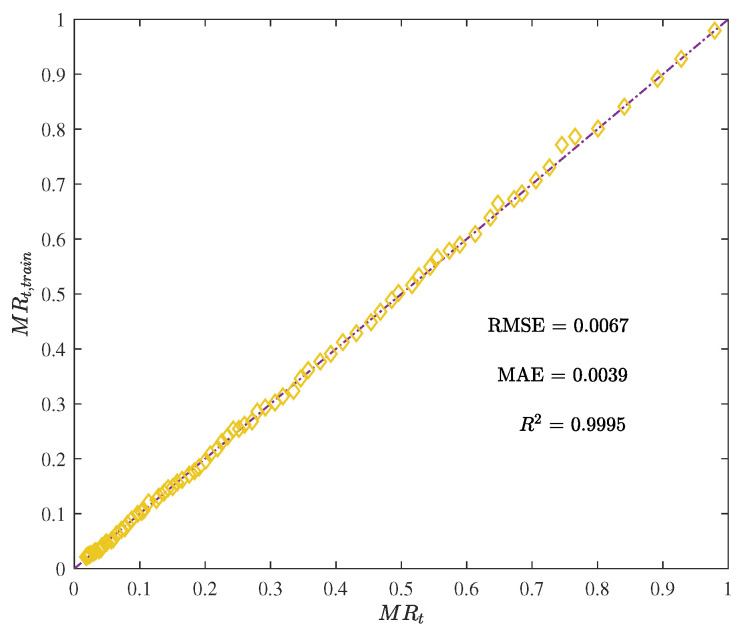
The moisture ratio given by the trained ANN model $MR_{t,train}$ vs. the actual moisture ratio $MR_{t}$.

Figure 5 and Figure 6 demonstrate the training results of the ANN, with RMSE, MAE, and $R^{2}$ reaching 0.0067, 0.0039, and 0.9995, respectively. These values indicate that the ANN had a low error rate and good performance on the training set.

**Figure 7:**
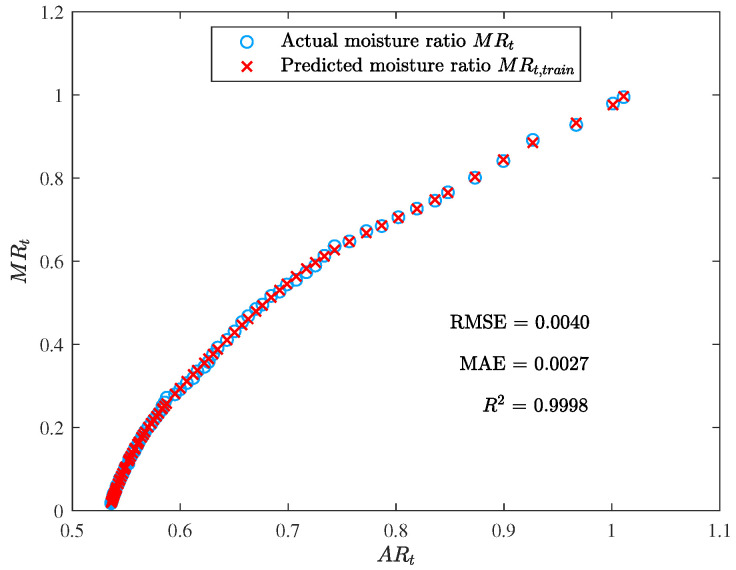
The training result of the ELM model (the moisture ratio given by the trained model $MR_{t,train}$ vs. the area ratio $AR_{t}$).

**Figure 8:**
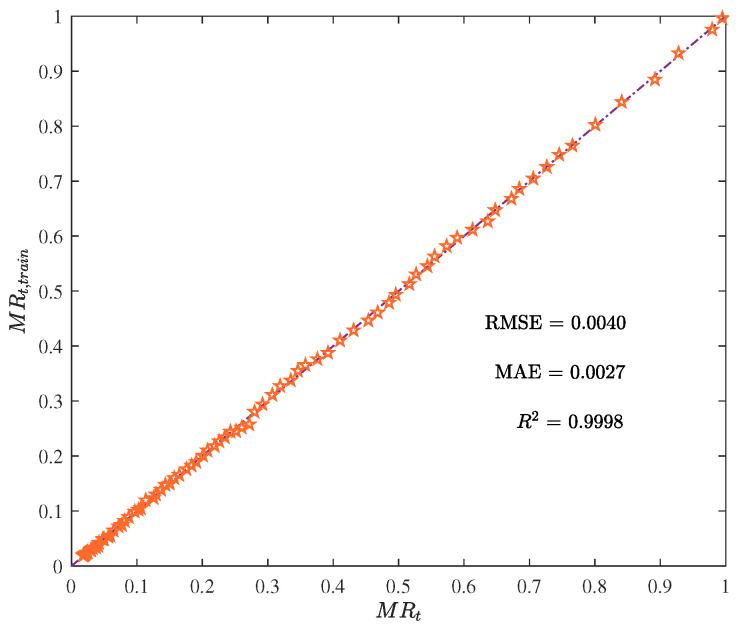
The moisture ratio given by the trained ELM model $MR_{t,train}$ vs. the actual moisture ratio $MR_{t}$.

Figure 7 and Figure 8 display the training outcomes of the ELM. The attained RMSE, MAE, and $R^{2}$ values of 0.0040, 0.0027, and 0.9998, respectively, reveal a high degree of accuracy and exceptional efficacy in modeling the training data.

### 3.2. Prediction

**Figure 9:**
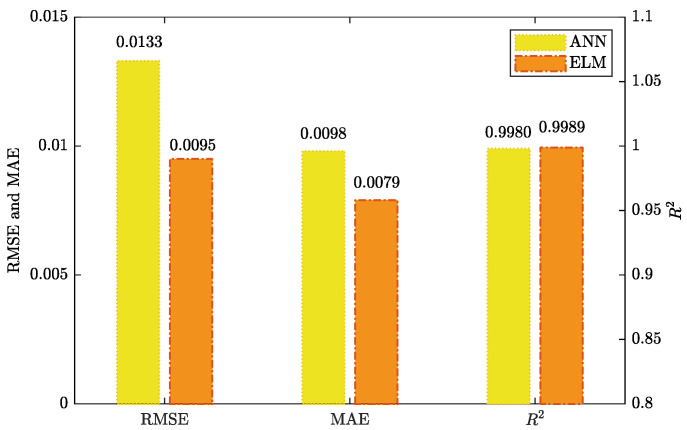
The performance comparison of prediction using ANN and ELM.

**Figure 10:**
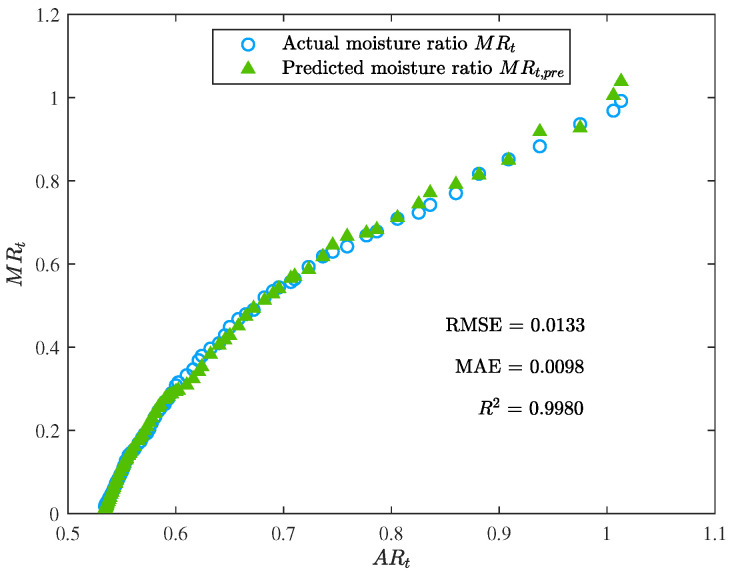
The prediction performance of the ANN model (the moisture ratio predicted by the ANN model $MR_{t,pre}$ vs. the area ratio $AR_{t}$).

**Figure 11:**
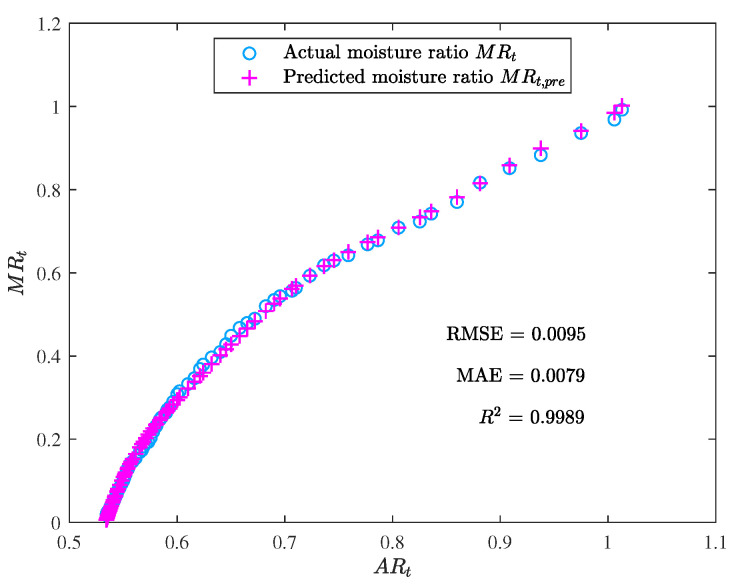
The prediction performance of the ELM model (the moisture ratio predicted by the ELM model $MR_{t,pre}$ vs. the area ratio $AR_{t}$).

**Figure 12:**
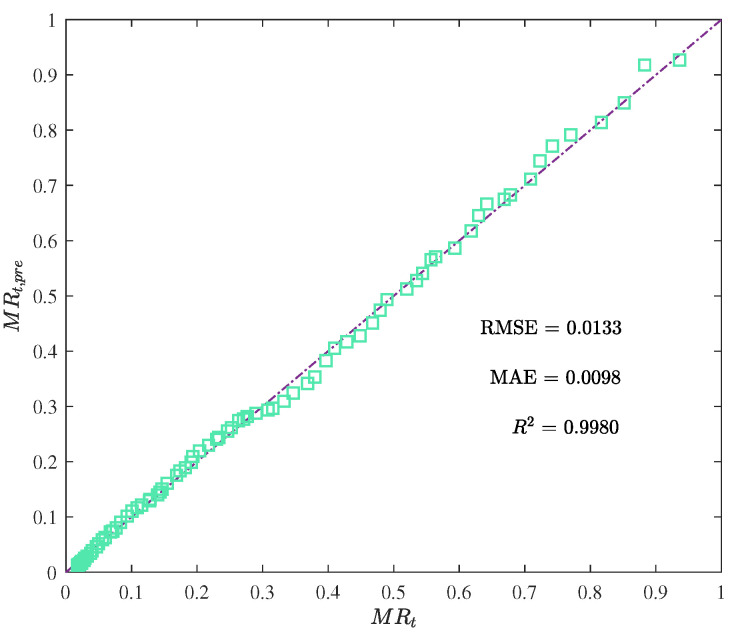
The moisture ratio predicted by the ANN model $MR_{t,pre}$ vs. the actual moisture ratio $MR_{t}$.

**Figure 13:**
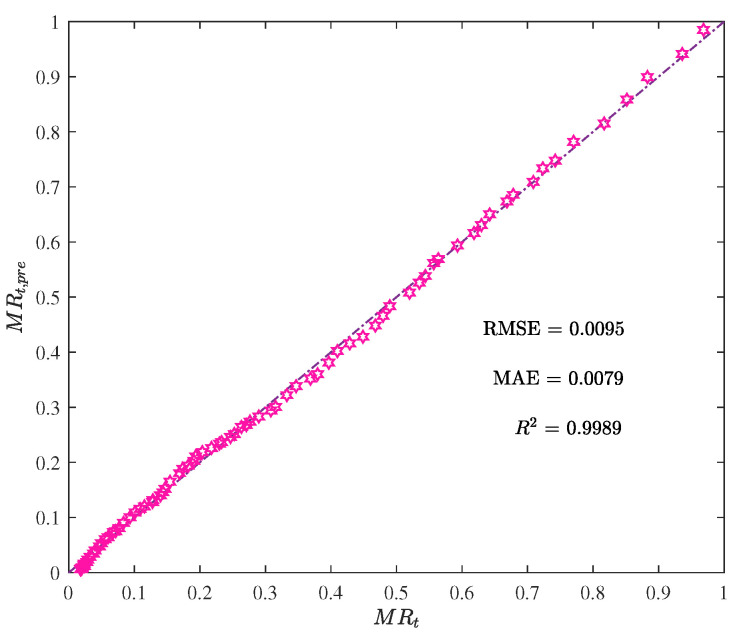
The moisture ratio predicted by the ELM model $MR_{t,pre}$ vs. the actual moisture ratio $MR_{t}$.

The performance metric comparison using ANN and EML for prediction is shown in Figure 9. Meanwhile, the prediction results of ANN and EML are displayed in Figure 10, Figure 11, Figure 12 and Figure 13.

The prediction results show that ELM outperformed ANN, while the latter’s performance was still acceptable. The main advantage of ELM lay in its training process, which was less sensitive to the selection of hyperparameters. In contrast, the performance of ANN heavily relied on the optimal selection of hyperparameters, such as the number of hidden layers and neurons, learning rate, and activation function. Although the resulting ANN model demonstrated good generalization ability, we observed a certain degree of overfitting during the training process. Additional techniques, such as weight decay and early stopping, had to be employed to overcome this issue.

In contrast, ELM had inherent antioverfitting properties, and its training process was less cumbersome. Moreover, compared to the ANN model, ELM had a significantly faster learning speed, which further supported the practicality of ELM for massive-scale data modeling.

In many prediction studies, the test set and training set data were selected from the same experiment, which limited the applicability of the model to other datasets. In contrast, in this experiment, a new dataset from a different experiment was used as the prediction set, which enhanced the model’s generalizability. The results demonstrated that ELM could be an effective means to predict changes in moisture content through material shrinkage, highlighting its potential as a useful tool in related applications.

## 4. Conclusions

A microwave drying system was developed to enable real-time image acquisition, weight measurement, and temperature measurement. An image-processing algorithm was devised to process the acquired images and derive area feature data. Constant-temperature microwave drying experiments were conducted, and the results demonstrated that the experimental system and algorithm could effectively obtain the area and moisture content of the materials in real time. ANN and ELM were utilized to model and predict moisture content, both of which achieved favorable results. However, ELM exhibited superior performance, a shorter training time, and better antioverfitting properties. Therefore, the combination of machine vision and ELM was a promising approach for predicting moisture content during the drying process.

## Data Availability

The data in this research are available upon request from the corresponding author.
